# Molecular evidence of *Babesia* in pet cats in mainland China

**DOI:** 10.1186/s12917-019-2214-0

**Published:** 2019-12-30

**Authors:** Xue-Lian Zhang, Xiao-Wen Li, Wen-Jun Li, Hui-Lan Huang, Shu-Jian Huang, Jian-Wei Shao

**Affiliations:** 1 Key Laboratory for Preventive Research of Emerging Animal Diseases, Foshan University, Foshan, 528231 Guangdong China; 2 College of Life Science and Engineering, Foshan University, Foshan, 528231 Guangdong China; 3 Department of Medical Microbiology and Parasitology, Zhejiang University School of Medicine, Zhejiang, 310058 Hangzhou China

**Keywords:** Molecular evidence, *Babesia*. *vogeli*, Pet cats, Mainland China

## Abstract

**Background:**

*Babesia* spp. are important emerging tick-borne protozoan hemoparasites, and pose a great impact on companion animals. Canine babesiosis has been well described worldwide, while felis babesiosis has primarily been reported from South Africa. To the best of our knowledge, *Babesia* spp. infections in dogs have been well elucidated in pet dog population in China, no report about *Babesia* spp. infection in cat population in mainland China.

**Results:**

In this study, a total of 203 blood samples were collected from pet cats in Shenzhen city, and detected the presence of *Babesia* spp. with nested-PCR. Sequence comparison based on the 18S rRNA gene and ITS region revealed that three cats (1.48%) were infected with *Babesia*. *vogeli*. Notably, the sequences of ITS region obtained in this study shared the highest nucleotide identity with the sequence of *B*. *vogeli* strain isolated in cat from Taiwan.

**Conclusions:**

This study is the first report about babesiosis in domestic cats, and also provides molecular evidence of *Babesia* spp. infection in cat in mainland China. The data present in this study suggest *B*. *vogeli* may be circulating in cat population in mainland China. Further study to investigate the epidemiology of *Babesia* infection in cat nationwide is warranted.

## Background

*Babesia* spp. are important emerging tick-borne protozoan hemoparasites with great economic, veterinary, and medical significance [1]. They are considered to be one of the most commonly found parasites in the blood of mammals, and they can naturally infect the red blood cells of a broad range of vertebrate hosts, including rodents, cattle, horses, humans, and companion animals (cats and dogs) [1]. Since the first description of in 1888 in Romanian cattle by Victor Babes, more than 100 *Babesia* species have been identified in wild and domestic animals [1]. In addition, ticks were identified as the mode of transmission of in 1893 [2, 3].

*Babesia* spp. pose a great impact on companion animals. Babesiosis caused by different *Babesia* species is a disease with a worldwide distribution characterized by erythrocyte destruction causing mild to severe systemic clinical manifestations [4]. Babesiosis in domestic cats was firstly report in India [5]. Then, sporadic cases of *Babesia* infection among domestic cats have been reported in different regions of the world, including Kenya, Venezuela, Israel, Brazil, Croatia, Poland, Thailand, Zimbabwe, France, and Germany [6–11]. However, babesiosis in cats has primarily been reported from South Africa, where infection is mainly due to *B*. *felis*, a small piroplasm that causes anemia and icterus [12]. Additionally, multiple species of *Babesia* have been documented in cats from Asia, Europe, and America [6, 13–19].

*Babesia* spp. infections in dogs have been well described in Shandong, Anhui, Zhejiang, Jiangsu, Jiangxi, Guangxi, Gansu, and Hubei Provinces of China, and demonstrated that *B*. *gibsoni* and *B*. *vogeli* were the causative agents of canine babesiosis [20–23]. Moreover, studies also suggested that the southern and eastern regions of China are the main endemic regions for *Babesia*, and *B*. *gibsoni* is the most widespread species in China, while *B*. *vogeli* is the other widespread species in dog population in China [20–23]. However, to the best of our knowledge, only one report about *B. hongkongensis*, which was discovered in kidney sections of a free-roaming cat in Hong Kong, has been described in China [19], and no information is available on the infection of *Babesia* spp. in cat population in mainland China.

## Results

### *Babesia* spp. detection

All DNA samples were subjected to screen the presence of *Babesia* spp. by nested PCR targeting the 18S rRNA gene of *Babesia* species. As shown in Table 1, out of the 203 blood samples screened, 3 positive samples for *Babesia* spp. infection were detected with an overall prevalence of 1.48% (3/203). The clinical records showed that the 3 positive pet cats (one female, Persian and two males, British Shorthair) were both under 1 year old and showed the clinical sign of fever. Other pet cats with overt clinical signs (fever, anorexia, or jaundice) were negative for *Babesia* spp. infection.

### Nucleotide sequence analysis

The full-length 18S rRNA and ITS region were amplified to further confirm the positive rates and better identify and characterize the *Babesia* spp. determined in this study.

Sequence similarity searches in BLAST revealed that the 3 newly sequences of 18S rRNA gene generated in this study were high similar (99% nucleotide identity) with the published sequences of *B*. *vogeli* available in GenBank. A closer comparison of these 3 sequences after alignment revealed that they shared 99.9% nucleotide identity among themselves and 99.93% nucleotide identity with the sequences of *B*. *vogeli* in dog from China (HM590440), Venezuela (DQ297390), Japan (AY077719, AB083374), and Brazil (AY371194, AY371195, AY371196).

Sequence similarity analysis based on the ITS region showed that the sequences generated in this study also shared high nucleotide identity with published ITS region sequences of *B*. *vogeli* (EF180054, EU084674, EU084675, EU084676, GQ395377). A closer comparison of ITS region sequences revealed that all the 3 newly sequences of ITS region shared the highest nucleotide identity (99.5–99.7%) with ITS region sequence of *B*. *vogeli* strain (EF180054) isolated from cat in Taiwan.

## Discussion

To the best of our knowledge, this study represents the first molecular confirmation of *Babesia* infection among cats in mainland China, and also indicated that these cats were infected with *B*. *vogeli*. Combined with Wong’s study conducted in Hong Kong [19], we assume that *Babesia* spp. is circulating in pet cat population in the southern region of China. Although *B*. *vogeli* has been detected in cats from Thailand by molecular method [17], no clinical features of feline babesiosis associated with *B*. *vogeli* infection have been described. In our study, the three positive pet cats detected by nested-PCR were both under 1 year old, which was consistent with previous studies have revealed that young animals are susceptible to *B*. *vogeli* infection [24]. More interestingly, the presence of *Babesia* species typical to dogs in domestic cats is detected sporadically by molecular methods often without compelling evidence of clinical infection [25].

In this study, we only collected blood samples from pet cats and observed only three (1.48%) samples positive for *Babesia* infection. Since pet cats sharing the better environment within households, spent most of their time indoors and they had limited chance to roam around and groom thoroughly. Thus, the positive rate of *Babesia* infection in pet cats was very low. Compare with the data obtained in the same areas of Shenzhen the frequency of pet dogs positive for *B*. *vogeli* infection in was (11.0%), which is significantly higher than that in pet cats (Li et al., unpublished data). This suggests that dogs may present the reservoir host and a better epidemiological sentinel for *B*. *vogeli* than cats.

Our study had some limitations. First, the positive rate in our study could not represent the actual infection rates since the blood samples were collected from pet cats. Second, no ticks were found from the body of pet cats, and we didn’t collect tick samples from the environment around the pet cats and detect the positive rate of *Babesia* spp. in ticks. However, the results present in our study indicate the presence of *Babesia* in cat population, and suggest that studies are needed to more fully investigate feline babesioses in Shenzhen.

## Conclusion

In conclusion, this study is the first report about babesiosis in domestic cats, and also provides molecular evidence of *Babesia* spp. infection in cat in mainland China. The data present in this study suggest *B*. *vogeli* may be circulating in cat population in other provinces of China. Further study to investigate the epidemiology of *Babesia* infection in cat nationwide is warranted.

## Methods

### Blood sample collection

**Fig. 1:**
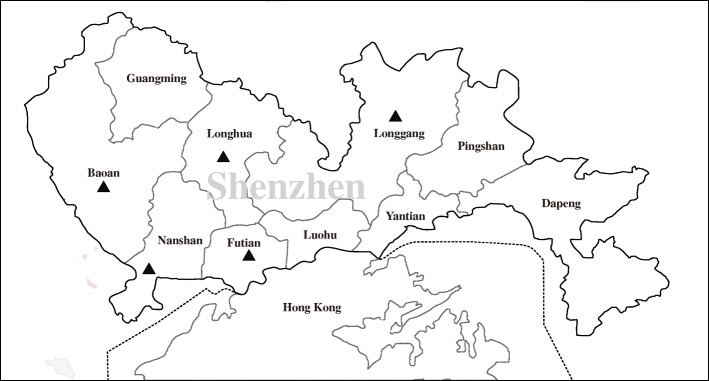
Map with the location of collecting sites of blood samples from pet cats (▲) in Shenzhen, China. It was drawn by us specific for this study, and plotted by combination of Surfer software version 4 (Golden Software, USA) and Photoshop CS 8.0.1 (Adobe Systems, USA)

From October 2018 to December 2018, a total of 203 blood samples were randomly collected from pet cats in local animal hospitals where located in five districts of Shenzhen (Fig. 1), and all samples have been screened the presence of *Bartonella* [26]. Blood samples were collected into EDTA-coated vacutainer tubes by veterinarians in local animal hospitals, and transported in dry ice to the laboratory of the College of Life Science and Engineering, Foshan University. The detailed information, including gender, number, and geographic distribution of these pet cats, is described in Table 1. These pet cats include 100 females and 103 males, and the age of them between 2 months and 3 years. Among these pet cats, 142 individuals were gone for vaccination, or for general inspection and without clinical sign, and 61 individuals presented with overt clinical presentations: fever, anorexia, or jaundice. No tick was found from the body of pet cats.

### DNA extraction and *Babesia* spp. detection

According to the suggesting protocol of Blood DNA Kit (Omega, Norcross, GA, USA), total DNAs were extracted from 200 μL of whole blood samples and then dissolved in 80 μL autoclaved double distilled water (ddH_2_O). The concentrations of the extracted DNA were measured with a NanoDrop 2000 (Thermo Scientific, USA), and DNA samples were stored at − 20 °C until further use.

Using the DNAs as templates, a nested PCR targeting the 18S rRNA gene of *Babesia* species was performed to detect the presence of *Babesia* spp. in the blood samples as previously described [20]. To prevent contamination, template isolation, PCR mixture preparation, template DNA addition, and agarose gel electrophoresis were performed in separate rooms, and dedicated pipets and tips with filter elements inside were used. The DNA sample of *B*. *gibsoni* extracted from dog blood, which presented by Dr. Guo from Northwest Agriculture & Forestry University, was used as the positive control, and double distilled water was used as negative control in all PCR amplification.

### Cloning and sequencing of full-length 18S rRNA and ITS region

To better identify and characterize the *Babesia* species determined in this study, the full-length 18S rRNA gene and internal transcribed spacers (ITS) region (including ITS1, 5.8S rRNA, and ITS2) were amplified from each positive sample with conventional PCR. The primer pairs described in previous study were used in the amplification of the full-length 18S rRNA gene, and the ITS region sequence [20]. The primer sequences and PCR parameters are shown in detail in Table 2.

**Table 1:** Prevalence of *Babesia* in pet cats in Shenzhen, China

Parameters	No. of cats tested (%)	Percentage (n) of infected cats	95% CI	*χ*^2^-value	*P*-value
Gender				0.309	0.578
Female	100 (49.3)	1.0 (1)	0.95–2.95		
Male	103 (50.7)	1.94 (2)	0.72–4.60		
Age				11.33	0.009
Juvenile (< 1 year)	43 (21.2)	7.0 (3)	0.63–14.6		
Adult (≥ 1 year)	160 (78.8)	0 (0)	–		
Health condition				7.088	0.026
Apparently healthy	142 (70.0)	0 (0)	–		
Sick	61 (30.0)	4.9 (3)	0.52–10.3		
Geographical location				5.597	0.231
Longgang	39 (19.2)	5.13 (2)	1.79–12.0		
Longhua	52 (25.6)	0 (0)	–		
Futian	26 (12.8)	0 (0)	–		
Nanshan	43 (21.2)	0 (0)	–		
Baoan	43 (21.2)	2.33 (1)	2.18–6.84

**Table 2:** Details of primers used in this study

gene	Primers	Sequences (5′ → 3′)	Tm	Amplicon (bp)	Reference
18S rRNA	Piro1-S	F1: CTTGACGGTAGGGTATTGGC	55 °C	1400	[27]
	Piro3-AS	R1: CCTTCCTTTAAGTGATAAGGTTCAC			[27]
	Piro-A	F2: ATTACCCAATMCBGACACVGKG	55 °C	407	[27]
	Piro-B	R2: TTAAATACGAATGCCCCCAAC			[27]
	P1	F: AACCTGGTTGATCCTGCCAGTAGTCAT	54 °C	1700	[20]
	P2	R:GATCCTTCTGCAGGTTCACCTAC			[20]
ITS region	ITS-F	F: GAGAAGTCGTAACAAGGTTTCCG	54 °C	1100	[20]
	ITS-2	R: ACAATTTGCGTTCAATCCCA			[20]

The PCR products with expected size amplified by each of the primer sets were purified using the QIAquick gel extraction kit (Qiagen, USA) according to the manufacturer’s recommendations, cloned into the pMD19-T vector (TaKaRa, China), which was then transformed into *E. coli* JM109 competent cells according to the manufacturer’s instructions. For each amplicon, the positive inserts were confirmed by PCR, and three positive clones were sent for sequencing by using universal M13 forward and reverse primers to the Sangon Biotechnology Company in China.

### Nucleotide sequence analysis comparison

The sequences generated in this study were assembled using the SeqMan program (DNASTAR, Madison, WI). All the newly generated sequences of both 18S rRNA gene and ITS region sequence were compared with each other and with published sequences in the nucleotide database in GenBank by BLAST program of the National Center for Biotechnology Information (NCBI: http://blas.ncbi.nlm.nih.gov) and MegAlign 7.0 software (DNAStar, USA) in order to analyze sequence variations.

### Statistical data analysis

Statistical Package for Social Sciences (SPSS) Version 22.0 was used to calculate the *P*-value with Chi-square or Fisher’s exact test to determine the differences of *Babesia* positive rates between sampling sites. A *P*-value < 0.05 was considered to be statistically significant.

## Data Availability

All sequences obtained in this study have been deposited in GenBank under the accession numbers MN067707-MN067709 and MN067711-MN067713.

## Abbreviations

*B*. *gibsoni*: *Babesia. gibsoni*
*B. hongkongensis*: *Babesia. hongkongensis*
*B*. *vogeli*: *Babesia. vogeli*
ITS: internal transcribed spacers
